# Cortisol Responses to Mental Stress and the Progression of Coronary Artery Calcification in Healthy Men and Women

**DOI:** 10.1371/journal.pone.0031356

**Published:** 2012-02-06

**Authors:** Mark Hamer, Romano Endrighi, Shreenidhi M. Venuraju, Avijit Lahiri, Andrew Steptoe

**Affiliations:** 1 Department of Epidemiology and Public Health, University College London, London, United Kingdom; 2 Clinical Imaging and Research Centre, Wellington Hospital, London, United Kingdom; California Pacific Medicial Center Research Institute, United States of America

## Abstract

**Background:**

Psychosocial stress is a risk factor for coronary heart disease (CHD). The mechanisms are incompletely understood, although dysfunction of the hypothalamic pituitary adrenal (HPA) axis might be involved. We examined the association between cortisol responses to laboratory-induced mental stress and the progression of coronary artery calcification (CAC).

**Methods and Results:**

Participants were 466 healthy men and women (mean age = 62.7±5.6 yrs), without history or objective signs of CHD, drawn from the Whitehall II epidemiological cohort. At the baseline assessment salivary cortisol was measured in response to mental stressors, consisting of a 5-min Stroop task and a 5-min mirror tracing task. CAC was measured at baseline and at 3 years follow up using electron beam computed tomography. CAC progression was defined as an increase >10 Agatston units between baseline and follow up. 38.2% of the sample demonstrated CAC progression over the 3 years follow up. There was considerable variation in the cortisol stress response, with approximately 40% of the sample responding to the stress tasks with an increase in cortisol of at least 1 mmol/l. There was an association between cortisol stress reactivity (per SD) and CAC progression (odds ratio = 1.27, 95% CI, 1.02–1.60) after adjustments for age, sex, pre-stress cortisol, employment grade, smoking, resting systolic BP, fibrinogen, body mass index, and use of statins. There was no association between systolic blood pressure reactivity and CAC progression (odds ratio per SD increase = 1.03, 95% CI, 0.85–1.24). Other independent predictors of CAC progression included age, male sex, smoking, resting systolic blood pressure, and fibrinogen.

**Conclusion:**

Results demonstrate an association between heightened cortisol reactivity to stress and CAC progression. These data support the notion that cortisol reactivity, an index of HPA function, is one of the possible mechanisms through which psychosocial stress may influence the risk of CHD.

## Introduction

The accumulating evidence that stress-related factors contribute to the development of cardiovascular disease (CVD) has stimulated research into the underlying pathways involved [1]. Psychophysiological stress testing can be used to better understand the mechanisms underlying the association between mental stress and CVD [2]. Existing work has largely focused on cardiovascular reactivity to stress as a tool to predict future risk, and the associations are modest but consistent [3]. The issue of whether stress reactivity contributes to the progression of underlying disease or only to the incidence of clinical cardiac events has led to research involving indicators of subclinical disease. For example, in 756 men from the Kuopio Ischemic Heart Disease study, systolic BP reactivity at the baseline assessment was related to carotid intima media thickness (IMT) after seven years follow up and also to the progression of IMT, independently of established risk factors [4]. Two separate studies in healthy women showed that cross-sectionally, greater pulse pressure and systolic BP reactivity were respectively associated with greater carotid IMT [5] and the presence of coronary artery calcium (CAC) [6].

The importance of stress reactivity in other biological pathways relevant to CVD risk has gained less attention. Abnormalities in hypothalamic pituitary adrenal (HPA) function have been described in several chronic inflammatory disorders, and may be a possible mechanisms through which psychosocial stress influences the risk of CVD [7], [8]. In a cross-sectional study containing healthy participants from the Whitehall II cohort we recently demonstrated an association between cortisol stress reactivity and subclinical coronary disease as indexed by CAC [9]. Since the interpretation of cross-sectional data can be problematic, we performed a prospective follow up of this study to examine the association between cortisol stress reactivity and CAC progression.

## Materials and Methods

### Participants

A sample of participants was drawn from the Whitehall II epidemiological cohort [10] for psychophysiological testing during 2006 to 2008 (baseline) and underwent scans at baseline and three years follow up to measure CAC progression. The criteria for entry into the study included no history or objective signs of CHD, no previous diagnosis or treatment for hypertension, inflammatory diseases, or allergies. Volunteers were of white European origin, aged 53–76 years, and 56.5% were in full-time employment. Selection was stratified by grade of employment (current or most recent) to include higher and lower socioeconomic status participants. Participants were prohibited from using any anti-histamine or anti-inflammatory medication 7 days before testing and were rescheduled if they reported colds or other infections on the day of testing. Participants gave full written informed consent to participate in the study and ethical approval was obtained from the UCLH committee on the Ethics of Human Research.

### Psychophysiological testing at baseline

Testing was performed in either the morning or afternoon in a light temperature-controlled laboratory, and was based on a protocol previously used in this laboratory [11]. Participants were instructed to refrain from drinking caffeinated beverages or smoking for at least 2 hours before the study and not to have performed vigorous physical activity or consumed alcohol the previous evening. After a 30 minute rest period baseline blood pressure (using an automated UA-779 digital monitor) and a saliva sample were taken. Two behavioral tasks, designed to induce mental stress, were then administered in a random order. The tasks were a computerized version of the Stroop task and mirror tracing, both of which have been used extensively in psychophysiological research [12]. The tasks each lasted for 5 minutes. Participants then rested for 75 minutes. Blood pressure measurements were continuously assessed throughout the protocol, using a Finometer device (Finapres Medical Systems, Amsterdam). Blood pressure reactivity was calculated from a pre-stress baseline period (average over 5 minutes) and mean blood pressure during the stressors aggregated across tasks. Saliva samples were collected immediately following the tasks and then at 20, 45, and 75 minutes post stress for the assessment of salivary cortisol. The samples were collected using Salivettes (Sarsted, Leicester, UK), which were stored at −30°C until analysis. Levels of cortisol were assessed using a time resolved immuno-assay with fluorescence detection, at the University of Dresden. The intra and inter-assay coefficients of variation were <8%. Peak responses in cortisol tended to occur immediately after the tasks, thus for the purposes of the present study samples at pre-stress and immediately following the tasks was used to calculate a stress response change score [Cort _change_ = Cort_post stress_−Cort_pre-stress_].

### Coronary artery calcification (baseline and follow up)

The assessment of CAC was performed at baseline and three years follow up using electron beam computed tomography (GE Imatron C-150, San Francisco, CA) as previously described [13]. In brief, 40 contiguous 3-mm slices were obtained during a single breath-hold starting at the carina and proceeding to the level of the diaphragm. Scan time was 100 ms/slice, synchronized to 40% of the R-R interval. Agatston and volumetric calcium scores were calculated to quantify the extent of CAC by a single experienced investigator blinded to the psychophysiological and clinical data on an Aquarius workstation (TeraRecon Inc., San Mateo, CA). Since calcified volume was very highly correlated with Agatston score (Spearman's r = 0.99), we present data for Agatston score only.

### Covariates

Participants reported current smoking levels. Height and weight were recorded in light clothing for the calculation of body mass index (BMI). Fasting blood samples were taken during a separate clinical assessment. Analysis of C-reactive protein (CRP) and fibrinogen was performed using high-sensitivity ELISA (R & D Systems, Oxford, UK). Total and high-density-lipoprotein (HDL) cholesterol and triglycerides was measured within 72 h in serum stored at 4°C using enzymatic colorimetric methods. Low-density-lipoprotein (LDL) cholesterol was derived using the Friedewald equation. Glucose homeostasis was assessed from glycated haemoglobin (HbA1C) concentration, assayed using boronate affinity chromatography, a combination of boronate affinity and liquid chromatography.

### Statistical analysis

We defined progression of CAC in two ways. Firstly, as a binary variable defined as change in Agatston score >10, a cut off previously used to assess significant progression in an asymptomatic population [14]. Secondly, in order to perform linear analysis we used a previously described formula [15], which expresses relative change in CAC as the change from baseline to follow in the log of CAC plus a constant [ln(CAC_fup_+25)−ln(CAC_baseline_+25)]. This approach has advantages over using percentage changes that are influenced by very small baseline CAC values for which a very small absolute increase can result in large percentage changes. The use of the log-scale also de-emphasizes very large follow-up CAC scores. Associations between cortisol reactivity (per SD change) and CAC progression were investigated using both logistic and linear regression, adjusting for age, gender, follow up time, pre-stress cortisol level, employment grade, use of statins, resting blood pressure, fibrinogen, HDL and LDL cholesterol, body mass index, and smoking. Associations between blood pressure reactivity and CAC progression were also performed using the approaches described above. All analyses were conducted using SPSS version 15.

## Results

At baseline 496 participants had complete data on cortisol, CAC, and all covariates. Thirty participants did not complete follow up (3 deceased, 7 lost to follow up, 20 declined), thus the analytic sample comprised 466 participants (mean age = 62.7±5.6 yrs; range, 53–76 yrs). Participants that did not complete follow up were slightly older than those that remained in the study (65.3 vs. 62.7 yrs, p = 0.01), although there was no difference in levels of baseline CAC (% without detectable CAC; 33.3 vs. 44.3%, p = 0.22). The average follow up period was 2.98 yrs, ranging from 1.75–3.55 yrs.

**Figure 1:**
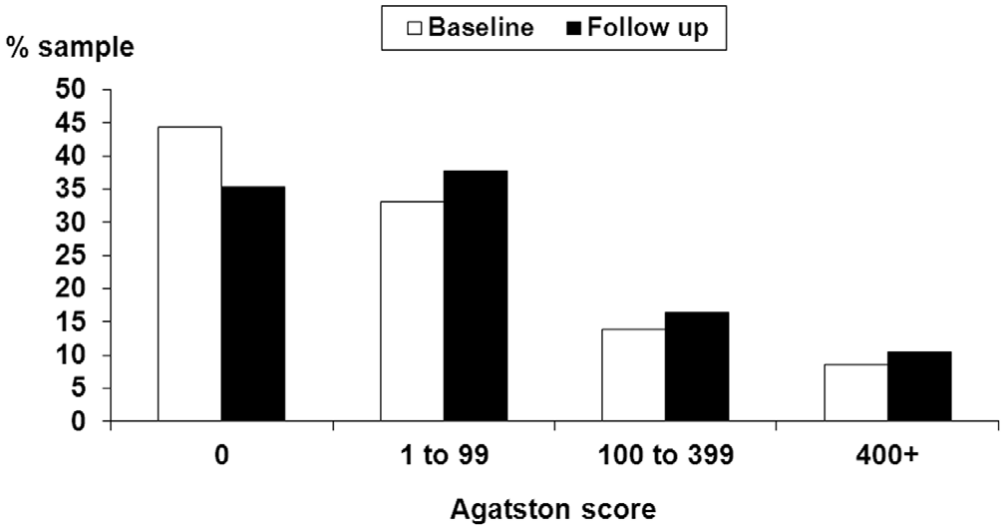
The progression of CAC over 3 years follow up (n = 466). Filled bars designate CAC scores at follow up. Coronary artery calcification scores of 100–400, 400–999, and ≥1000 predict up to 4-, 7-, and 11-fold increases in future CVD risk, respectively, compared with patients with no detectable CAC. There was a significant increase in log Agatston score between baseline and follow up (2.25 vs 2.65, p<0.001), and 38.2% had progression of CAC as defined by an increase in >10 Agatston units.

Among participants with no detectable CAC at baseline (n = 215, 44.3% of sample), 50 of them developed new incident CAC during the 3 year follow up (see Figure 1). In the overall sample there was a significant increase in log Agatston score between baseline and follow up (2.25 vs 2.65, p<0.001), and 38.2% had progression of CAC as defined by an increase in >10 Agatston units. Participants with CAC progression >10 units were older, more likely to be male, smokers, statin users, have higher blood pressure, lower HDL cholesterol, and higher fibrinogen levels compared to participants without CAC progression (Table 1).

**Table 1:** Characteristics of the study population at baseline in relation to CAC progression (N = 466).

Variable	No progression (N = 288)	CAC progression* (N = 178)	p-value
Age (yrs)	62.3±5.3	63.3±5.9	0.046
Men (%)	135 (46.9)	118(66.3)	<0.001
Highest work grade (%)	116 (40.3)	73 (41.0)	0.88
Current smokers (%)	11 (3.8)	14 (7.9)	0.06
Resting systolic BP (mmHg)	121.3±16.5	126.8±15.7	<0.001
HDL cholesterol (mmol/l)	1.74±0.48	1.62±0.44	0.01
LDL cholesterol (mmol/l)	3.89±0.96	3.94±0.91	0.56
C-reactive protein (mg/l)	1.59±2.07	1.60±2.07	0.94
Fibrinogen (g/l)	3.08±0.59	3.26±0.62	0.002
Body mass index (kg/m^2^)	25.7±4.2	26.1±3.6	0.29
HbA1c (%)	5.46±0.39	5.44±0.38	0.62
Pre-stress cortisol (mmol/l)	6.58±4.29	6.52±4.81	0.88
Statins use (%)	21 (7.3)	22 (12.4)	0.07
Mean follow up (days)	1081±81	1104±74	0.003

*CAC progression defined as an increase >10 Agatston units between baseline and follow up.

Values are means ± SD.

There was considerable variation in the cortisol stress response, with approximately 40% of the sample responding to the stress tasks with an increase in cortisol of at least 1 mmol/l [9]. In analysis adjusted for age and sex, the cortisol stress response (per SD increase) was associated with CAC progression (odds ratio = 1.26, 95% CI, 1.02–1.52). In further models the association remained unaltered (odds ratio = 1.27, 95% CI, 1.02–1.60) after additional adjustments for follow up time, pre-stress cortisol level, employment grade, use of statins, resting blood pressure, fibrinogen, HDL and LDL cholesterol, body mass index, and smoking. Other independent predictors of CAC progression included age, male sex, smoking, systolic blood pressure, and fibrinogen (Table 2).

**Table 2:** Risk factors for CAC progression of >10 Agatston units (N = 466).

*Risk factor*	Odds Ratio (95% CI)*
Age (per yr)	**1.05 (1.01–1.10)**
Male sex	**2.11 (1.23–3.63)**
Cortisol stress response†	**1.27 (1.02–1.60)**
Resting Systolic BP†	**1.25 (1.01–1.56)**
Fibrinogen†	**1.47 (1.20–1.81)**
Smoking	**2.50 (1.06–5.89)**
LDL cholesterol†	1.02 (0.82–1.28)
HDL cholesterol†	0.76 (0.45–1.29)
C-Reactive protein†	0.99 (0.89–1.10)
Body mass index†	1.02 (0.82–1.28)
HbA1c†	0.86 (0.49–1.48)
Statins use	1.50 (0.76–2.96)

*Effect estimates are mutually adjusted for all presented variables and follow up time.

† per SD increase.

We also performed linear analyses to examine the associations between cortisol and relative CAC change. In these analyses, CAC change was associated with age, male sex, systolic BP, fibrinogen, and cortisol reactivity (Table 3). In further multivariate models, including adjustments for age, sex, pre-stress cortisol, employment grade, smoking, resting systolic BP, fibrinogen, lipids, body mass index, and use of statins, the association of cortisol reactivity was marginally attenuated but remained independently associated with relative change in CAC (B = 0.011, 95% CI, −0.001–0.023, p = 0.066). In analyses stratified by the presence of CAC at baseline, the association between cortisol response and CAC change was only evident in participants without detectable CAC at baseline (fully adjusted B = 0.017, 95% CI, 0.006–0.027, p = 0.002).

**Table 3:** Risk factors for relative CAC changes over follow up (N = 466).

*Risk factor*	Coefficient* (95% CI)	P-value
Age	0.005 (0.00–0.01)	0.035
Male sex	0.060 (0.007–0.113)	0.026
Resting Systolic BP†	0.036 (0.009–0.062)	0.009
LDL cholesterol†	0.005 (−0.021–0.032)	0.724
HDL cholesterol†	−0.002 (−0.030–0.027)	0.901
C-Reactive protein†	−0.005 (−0.031–0.021)	0.716
Fibrinogen†	0.040 (0.014–0.067)	0.003
Body mass index†	0.009 (−0.018–0.035)	0.515
HbA1c†	−0.017 (−0.044–0.010)	0.214
Statins use	0.013 (−0.078–0.105)	0.775
Pre-stress cortisol	−0.001 (−0.007–0.005)	0.782
Cortisol reactivity†	0.010 (0.000–0.021)	0.049
Cortisol AUC	−0.002 (−0.028–0.025)	0.902

*Effect estimates are adjusted for age and sex.

† per SD increase.

BP – Blood pressure; HbA1c - glycated haemoglobin; AUC - Area under the curve.

On average, systolic blood pressure increased by 30.8±15.1 mmHg (range = −6.1 to 85.3 mmHg) in response to the stress tasks, and there was a significant correlation between cortisol stress reactivity and systolic blood pressure reactivity (*Pearson r* = 0.15, p = 0.001). However, systolic blood pressure reactivity was not associated with CAC progression (age and sex adjusted odds ratio per SD increase = 1.03, 95% CI, 0.85–1.24), and neither was diastolic blood pressure reactivity (age and sex adjusted odds ratio per SD increase = 0.96, 95% CI, 0.80–1.16). Systolic blood pressure reactivity was similar in participants with and without detectable CAC at baseline, respectively (31.8±15.8 vs. 30.2±14.2 mmHg, p = 0.43), and also similar in those who did and did not have CAC progression (31.5±15.8 vs. 30.2±14.6 mmHg, p = 0.34).

## Discussion

Previous data have shown associations between heightened cardiovascular reactivity and future CVD risk, although the utility of biological stress responses in predicting risk have not been adequately examined. The aim of this study was to investigate the association between cortisol stress reactivity and the progression of sub-clinical coronary atherosclerosis in healthy men and women. We observed an association between cortisol reactivity and CAC progression, with a 27% increase in the odds of progression per SD change in cortisol responsivity. These associations were largely independent of conventional risk factors. This relationship was most evident in participants without detectable CAC at baseline, which further supports the notion that heightened cortisol reactivity might be important in the aetiology of atherosclerosis and is not simply a marker of disease progression. Indeed, the development of new incident CAC reflects a different stage of the disease process compared with increases in existing calcification.

To our knowledge, this is the first study to show a prospective association between cortisol stress reactivity and progression of sub-clinical atherosclerosis. Other studies have examined associations between sympathetic nervous activity and various CVD risk factors. For example, in a small prospective study conducted on Norwegian military personal, norepinephrine responses to mental stress and cold pressor at the baseline examination was associated with insulin resistance and blood pressure at the 18 year follow up assessment [16], [17]. Several population studies have demonstrated associations between diurnal cortisol patterns and CVD; Dekker et al [18] observed an association between total cortisol exposure while awake and higher carotid plaque scores in a sample of older adults, whilst another study showed a greater presence of CAC in younger participants with a flatter diurnal cortisol decline [19]. Also, a flatter slope in cortisol levels across the day was associated with an increased risk of CVD mortality in British civil servants [20], and 24 hr urinary cortisol was associated with CVD death in the InCHIANTI prospective cohort study of older participants [21]. Several studies have also linked raised cortisol levels with metabolic risk factors, including fasting glucose, lipids, and obesity [22], [23]. The findings from clinical patient groups are less clear. Low serum cortisol levels were shown to predict death following myocardial infarction [24] and reduced cortisol stress responses in patients with stable CAD have also been observed [25]. In contrast, elevated fasting cortisol was associated with risk of future cardiac events in patients with chronic heart failure [26], [27]. Interestingly, in our study CAC progression was unrelated to resting cortisol levels or total cortisol production (area under the curve) over the psychophysiological testing period. The equivocal nature of some of these findings might be related to the strong diurnal cortisol pattern that can heavily influence the results. Therefore, single measures of cortisol might not be appropriate to capture the dynamic nature of HPA activity. In this regard, psychophysiological testing is advantageous since extrinsic factors can be tightly controlled.

Previous work has demonstrated that heightened blood pressure responses to laboratory induced stressors is associated with CVD risk, such as progression of IMT and hypertension [3], [4]. In a young, healthy sample of women, aged 20 to 35 years at baseline, each 10 mm Hg change in systolic blood pressure during a video game stressor was associated with a 24% increased odds of having CAC after 13 years follow-up, although there was no association with blood pressure reactivity during a star tracing task [6]. Thus, our null findings on blood pressure responses and CAC are inconsistent with some previous work in this area. Nevertheless, our sample was considerably older than in many previous studies that might partly account for the findings. In addition, previous blood pressure reactivity studies have only collected CAC measures at one point in time and were thus unable to examine CAC progression. Taken together, the different effects of blood pressure and cortisol reactivity on CAC progression shown here highlight the importance of examining both cardiovascular and neuroendocrine indices of stress reactivity in psychophysiological studies.

Relatively few studies have examined risk factors for the progression of CAC. In one of the largest to date, 5756 participants from the Multi-Ethnic Study of Atherosclerosis (MESA) were followed up over 2 years, and results showed that most traditional CVD risk factors were associated with both the risk of developing new incident CAC and increases in existing calcification [15]. However, low and high density lipoprotein cholesterol was only predictive of new incident CAC in MESA. These findings might partly reflect differences in the definition of CAC progression. For example, in the present study LDL cholesterol was the only risk factor associated with new incident CAC (data not shown), although cortisol, smoking, blood pressure and fibrinogen were associated CAC progression when defined as an increase of >10 Agatston units. This might also reflect differences in the mechanisms involved at various stages of the atherosclerotic process.

The mechanisms by which HPA activity directly influences atherosclerosis remain poorly understood, although there is some evidence that increased circulating cortisol levels may promote perivascular inflammation [28], and treatment with glucocorticoids has been shown to enhance calcification within arteriosclerotic lesions [29]. A previous study in healthy participants demonstrated that mental stress-induced endothelial dysfunction and baroreflex impairment was prevented by blocking cortisol production with metyrapone [30]. Thus, heightened cortisol responses may to some extent drive changes in hemodynamic function. Others have reported reduced cortisol stress responses in patients with stable CAD, and suggested that cortisol might act as a powerful anti-inflammatory agent in preventing atherosclerotic processes [8], [25]. In the present study, however, we did not observe any associations between cortisol reactivity and markers of inflammation as indexed by C-reactive protein [9]. We cannot, however, rule out the role of unmeasured confounding risk factors or genetic influences that might account for cortisol response patterns [31] and CHD risk [32]. For example, recent evidence suggests that a common glucocorticoid receptor polymorphism is related to higher pro-inflammatory activity and greater risk of CHD [32].

The present study has a number of strengths and limitations. The major strength is the prospective design of the study that allows greater confidence in interpreting the directionality of the observed relationships. The findings add to the evidence that stress-related processes are associated not only with the occurrence of clinical CVD, but also with progression of underlying coronary disease development. It should be noted that there was a large amount of variability in individual responses to the stressors, and only 40% of participants in this study were defined as cortisol responders, which is consistent with our previous findings from another sample tested with the same behavioural tasks [33]. Cortisol responses to stress tend to be greater when participants are confronted by social-evaluative challenges, rather than psychomotor and problem-solving tasks of the type used here. Cortisol stress responses were measured on a single occasion, and there may be adaptation on repeated testing, although we have previously demonstrated strong reproducibility of these responses over two repeated stress sessions [34].

In conclusion, we have demonstrated a prospective association between cortisol responses to laboratory-induced mental stress and CAC progression. These findings provide support for the hypothesis that hyper-reactivity of the HPA axis is one of the mechanisms through which psychosocial stress may influence the risk of CHD.
